# Exploration of Aberrant E3 Ligases Implicated in Alzheimer’s Disease and Development of Chemical Tools to Modulate Their Function

**DOI:** 10.3389/fncel.2021.768655

**Published:** 2021-11-18

**Authors:** Frances M. Potjewyd, Alison D. Axtman

**Affiliations:** Division of Chemical Biology and Medicinal Chemistry, Structural Genomics Consortium, UNC Eshelman School of Pharmacy, Chapel Hill, NC, United States

**Keywords:** Alzheimer’s disease, neurodegeneration, E3 ligase, chemical tools, structures, ubiquitination, PROTAC, proteolysis targeting chimera

## Abstract

The Ubiquitin Proteasome System (UPS) is responsible for the degradation of misfolded or aggregated proteins via a multistep ATP-dependent proteolytic mechanism. This process involves a cascade of ubiquitin (Ub) transfer steps from E1 to E2 to E3 ligase. The E3 ligase transfers Ub to a targeted protein that is brought to the proteasome for degradation. The inability of the UPS to remove misfolded or aggregated proteins due to UPS dysfunction is commonly observed in neurodegenerative diseases, such as Alzheimer’s disease (AD). UPS dysfunction in AD drives disease pathology and is associated with the common hallmarks such as amyloid-β (Aβ) accumulation and tau hyperphosphorylation, among others. E3 ligases are key members of the UPS machinery and dysfunction or changes in their expression can propagate other aberrant processes that accelerate AD pathology. The upregulation or downregulation of expression or activity of E3 ligases responsible for these processes results in changes in protein levels of E3 ligase substrates, many of which represent key proteins that propagate AD. A powerful way to better characterize UPS dysfunction in AD and the role of individual E3 ligases is via the use of high-quality chemical tools that bind and modulate specific E3 ligases. Furthermore, through combining gene editing with recent advances in 3D cell culture, *in vitro* modeling of AD in a dish has become more relevant and possible. These cell-based models of AD allow for study of specific pathways and mechanisms as well as characterization of the role E3 ligases play in driving AD. In this review, we outline the key mechanisms of UPS dysregulation linked to E3 ligases in AD and highlight the currently available chemical modulators. We present several key approaches for E3 ligase ligand discovery being employed with respect to distinct classes of E3 ligases. Where possible, specific examples of the use of cultured neurons to delineate E3 ligase biology have been captured. Finally, utilizing the available ligands for E3 ligases in the design of proteolysis targeting chimeras (PROTACs) to degrade aberrant proteins is a novel strategy for AD, and we explore the prospects of PROTACs as AD therapeutics.

## Introduction

### The Role of E3 Ligases in the Ubiquitin Proteasome System

**FIGURE 1:**
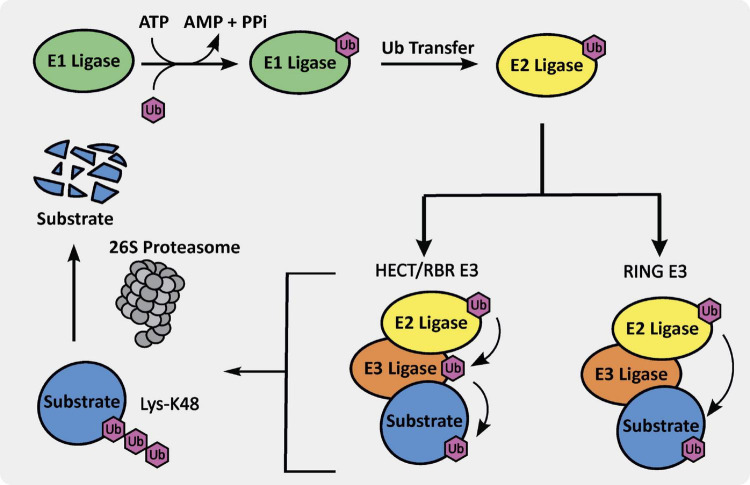
The ubiquitin proteasome system (UPS) cascade.

Through tagging of proteins with ubiquitin to induce degradation, the UPS helps with maintaining protein homeostasis. This process begins with the activation of ubiquitin via ATP conversion to AMP and resultant attachment of ubiquitin to an E1 ligase, which is a ubiquitin-activating enzyme (Figure 1). The E1 ligase then transfers the ubiquitin to an E2 ligase, which is a ubiquitin-conjugating enzyme, and binds to a specific E3 ligase, generating an E2-E3-Ub complex. This E2-E3-Ub complex is primed for transfer of ubiquitin to a specific protein that binds to a particular E3 ligase. If the target protein is tagged via K48-linked ubiquitin, it is brought to the 26S proteasome and degraded. This process is essential for maintaining protein homeostasis and an integral part of normal cell function and survival (Jana, 2012).

E3 ubiquitin ligases are integral members of the UPS. There are three different E3 ubiquitin ligase classes, including really interesting new gene (RING), homologous to E6AP C-terminus (HECT), and ring-in-between-ring (RBR) E3 ligases, which are further subdivided into different sub-classes (Morreale and Walden, 2016). As shown in Figure 1, the RING E3 ligase ubiquitin transfer mechanism is arguably the simplest and involves ubiquitin transfer directly from the E3-E2-Ub complex to a lysine residue on the bound substrate. There are approximately 600 RING E3 ligases in humans, which are subdivided into monomeric, homodimeric, heterodimeric, monomeric U-box, homodimeric U-box, and cullin-RING types. The HECT and RBR E3 ligases ubiquitin transfer mechanism requires a primary transthiolation from a cysteine on the E2 ligase to a cysteine on the E3 ligase domain prior to transfer to the substrate (Figure 1). There are approximately 30 HECT E3 ligases in humans, divided into the neuronal precursor cell expressed developmentally downregulated 4 (NEDD4) and HECT and RLD domain containing (HERC) families, primarily. Finally, there are only approximately 12 RBR E3 ligases, including proteins such as Parkin, making it the smallest sub-class.

### Mechanism of E3 Ligase-Mediated Dysfunction in Alzheimer’s Disease

While the UPS is active during normal neurodevelopment, aberrant functioning of E3 ligases as part of the UPS has been suggested to drive neurodegeneration (Upadhyay et al., 2017). The ability of small molecule ligands to bind to E3 ligases provides a promising strategy to elicit either inhibition or activation and correct the mis-regulation that can occur (Gong et al., 2016). When the function of the UPS is impaired, degradation of either misfolded or damaged proteins may not occur, leading to aberrant cellular functions (Jana, 2012). Specifically in the case of AD, the UPS dysfunction plays a key role in the regulation of synaptic plasticity and transmission, regulation of mitochondria, autophagic flux, and endoplasmic reticulum (ER) homeostasis (Tydlacka et al., 2010).

As essential proteins involved in neuronal homeostasis and signaling are disrupted when the UPS is dysfunctional in AD, aberrant synaptic activities and pathways that result in neuronal death are propagated in AD patients. Synaptic function is often reduced in AD due to accumulation of misfolded and hyperphosphorylated-tau and this tau pathology results in neuronal apoptosis (Colom-Cadena et al., 2020). Zhou et al. (2017) demonstrated in rat and fly neurons that pathogenic frontotemporal dementia and parkinsonism can be linked to chromosome 17 (FTDP-17) mutant tau. FTDP-17 mutant tau forms microtubules in the axons of neurons, is localized to presynaptic sites, binds to synaptic vesicles, and impairs both synaptic vesicle mobility and release rate (Zhou et al., 2017). Soluble Aβ peptides and Aβ oligomers (AβO) that bind to synaptic receptors are linked to impairment of synaptic plasticity (Viola and Klein, 2015). Additionally, hyperactive microglial and neuroinflammatory mechanisms also play a role in synaptic dysfunction. Activated microglia can cause synaptic loss and release pro-inflammatory kinases, resulting in synaptic toxicity (Jackson et al., 2019). E3 ligases that are implicated in synaptic loss and/or dysfunction include ubiquitin protein ligase E3A (Ube3A) and NEDD4-1 (Rodrigues et al., 2016; Olabarria et al., 2019).

Likewise, impaired mitochondrial function in AD is made worse by E3 ligases that are not functioning properly. Based on analysis of post-mortem human brain tissue from the dorsolateral prefrontal cortex (BA41/42) of individuals with AD pathology, it was reported that AD results in reduced mitochondrial expression at presynaptic terminals within this brain region (Pickett et al., 2018). Furthermore, primary hippocampal neurons treated with AβO demonstrated less axonal trafficking of mitochondria toward synapses in an *N*-methyl-D-aspartate (NMDA) receptor-dependent manner (Decker et al., 2010). Damaged mitochondria are degraded via the PTEN induced kinase 1 (PINK1)/Parkin E3 ubiquitin ligase mediated pathways, by which mitochondrial outer membranes are ubiquitinated for mitophagic degradation (Wang W. et al., 2020). Mitochondria targeted by Parkin are believed to accumulate in the somatodendritic region of neurons and await lysosomal degradation (Cai and Jeong, 2020). Parkin-mediated mitophagy is activated by Aβ accumulation in mutant human amyloid precursor protein (hAPP)-expressing transgenic neurons and in AD patient brains, and a disease-related decrease in cytosolic Parkin results in mitophagy dysfunction (Ye et al., 2015).

E3 ligases also play roles in autophagy that are disrupted in AD. Among other roles, autophagy is a key process that mediates the clearance of misfolded and/or aggregated proteins (Uddin et al., 2018). Bordi et al. (2016) evaluated CA1 hippocampal pyramidal neurons from early and late-stage AD patients and reported reduced autophagic flux. This finding was based on the observations of poor substrate clearance, accumulation of promoters of autophagic degradation such as LC3-II and SQSTM1/p62 in autolysosomes, and changes in autolysosomal size and area. Long et al. (2020) demonstrated that in AD patient brains as well as in animal and cell models, Aβ can activate autophagy and mutation of the APP gene to APPswe (APP695 Swedish mutation) disrupts the fusion of autophagosomes with lysosomes, leading to impaired autophagy. E3 ligases that are implicated in autophagy include, but are not limited to Parkin, TNF receptor associated factor 6 (TRAF6), and NEDD4-1 (Kuang et al., 2013).

A final role of E3 ligases is related to the ER, which does not function properly in AD. One of the functions of the ER is the removal of misfolded proteins. ER stress in AD is linked to inflammatory pathways that interrupt this essential ER function, allowing protein aggregates to accumulate (Salminen et al., 2009). ER stress markers have been characterized in neurons and not glial cells, and prolonged ER stress can lead to synaptic loss and axonal degradation (Salminen et al., 2009). The E3 ligase HMG-CoA Reductase Degradation 1 Homolog (HRD1) is expressed in the ER membrane of brain neurons and is responsible for ER-associated degradation (ERAD), a process that will be discussed in more detail in a subsequent section devoted to HRD1 (Zheng et al., 2016).

### Targeting E3 Ligases With Small Molecules

E3 ligase ligands are important in the design and preparation of PROTACs, which are heterobifunctional molecules containing a ligand for an E3 ligase and a protein of interest (POI) that is targeted for proteasomal degradation. The PROTAC brings the E3 ligase into close proximity with the POI, forms a ternary complex, ubiquitinates a lysine residue on the POI, and elicits proteasomal degradation of the POI. Despite more than 600 known E3 ligases, only approximately 10 small molecule E3 ligase ligands of varying quality have been characterized (Ishida and Ciulli, 2021). Within the PROTAC field only two of these E3 ligases are routinely targeted, Von-Hippel Lindau (VHL) and cereblon (CRBN), since small molecule ligands have been identified (Fischer et al., 2014; Galdeano et al., 2014). Discovery of ligands for additional E3 ligases that can be recruited to ubiquitinate target proteins will enable development of PROTACs that induce protein degradation. Additionally, tissue- or disease-specific E3 ligases that are upregulated in AD patient brains could be targeted to localize the degradation. Furthermore, development of E3 ligase ligands with improved physicochemical properties could increase PROTAC cell permeability and possibly enable delivery to the brain.

The structural differences and complexities of E3 ligases preclude a one-size-fits-all approach for small molecule inhibitor or activator development. Additionally, the current paucity of chemical modulators available for E3 ligases includes several with poor physicochemical properties, suboptimal potency, and often unexplored selectivity. Development of chemical probe quality molecules is of high importance to enable unambiguous characterization of the role(s) that these proteins play in a cellular context (Frye, 2010; Arrowsmith et al., 2015). Herein, we review the specific E3 ligases implicated in AD with altered expression contributing to disease progression. We next provide an overview of the structural data available for these E3 ligases and published exogenous ligands. This information will be invaluable in any campaign directed at targeting E3 ligases as a method of therapeutic intervention for AD. To fill the gaps in this knowledge, several current strategies that are needed to pursue E3 ligase ligand discovery are summarized. Finally, the promising potential of the use of PROTACs for the treatment of AD is introduced as a future direction that is enabled by the knowledge and technologies highlighted in other chapters of this review.

## E3 Ligases With Expression Changes in Alzheimer’s Disease

### Upregulated or Overactive E3 Ligases

**FIGURE 2:**
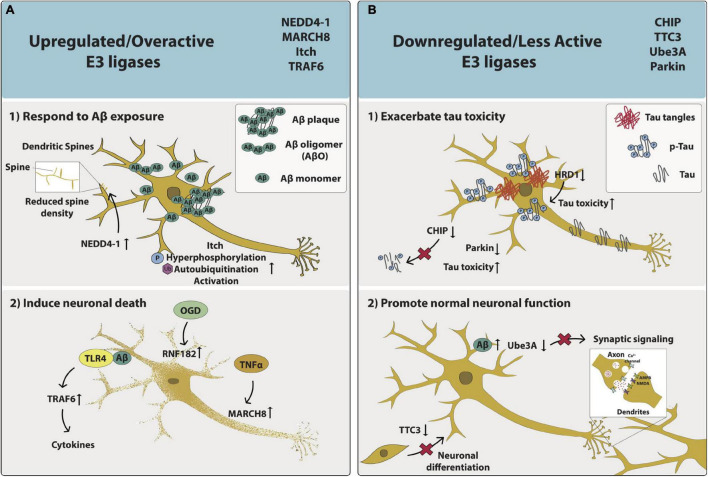
The phenotypic effects of aberrant E3 ligase function on AD neurons. The impacts of upregulated/overactive E3 ligases NEDD4-1, MARCH8, Itch and TRAF6 as well as downregulated/less active E3 ligases CHIP, TTC3, Ube3A, and Parkin on neuronal function and/or health are captured specifically within the context of AD. **(A)** Upregulated/overactive E3 ligases – **(1)** respond to Aβ exposure: NEDD4-1 relocates to dendritic spines and is overexpressed, leading to decreased spine density. Itch is hyperphosphorylated, autoubiquitinated, and becomes activated; or **(2)** induce neuronal death: Aβ interacts with TLR4, activates TRAF6, and results in cytokine release. OGD increases RNF182 expression. TNFα increases MARCH8 expression. **(B)** Downregulated/less active E3 ligases **(1)** exacerbate tau toxicity: Tau expression leads to reduced CHIP expression and less CHIP-induced tau elimination. HRD1 expression is decreased and p-Tau expression is increased. Parkin expression is decreased; or **(2)** promote normal neuronal function: TTC3 expression is decreased and neuronal differentiation is disrupted. Aβ levels are increased and Ube3A levels are decreased, leading to synaptic loss.

Either upregulation or overactivity has been observed with respect to certain E3 ligases in AD patients (Figure 2 and Table 1). The overactivation of E3 ligases can occur via a variety of complex mechanisms dependent on the type of E3 ligase. These mechanisms include gain-of-function mutations, phosphorylation-induced autoubiquitination, and binding of adaptor proteins, among others (Buetow and Huang, 2016). Increased activity of an E3 ligase results in enhanced degradation of native targets. Examples of E3 ligases that are marked by unusually elevated expression or activity in AD include NEDD4-1, Itchy homolog (Itch), TRAF6, and ring finger protein 182 (RNF182), and membrane-associated RING-CH (MARCH8). It is suggested that small molecules could be used to inhibit the function or to degrade these aberrant E3 ligases with potentially therapeutically beneficial results for AD patients.

**TABLE 1:** Summary of E3 ligases identified to have aberrant function(s) in AD, corresponding AD phenotypes, published ligands, and solved structures.

Status in AD	E3 ligase	AD phenotype	Ligands identified	Structures (PDB ID)
Upregulated	NEDD4-1	Synaptic plasticity and function (Kwak et al., 2012; Rodrigues et al., 2016).	Covalent compound 1 (*K*_*I*_ = 245.8 μM), covalent compound 2, covalent compound 3 (*K*_*I*_ = 29.3 μM) (Figure 3) (Kathman et al., 2015).	4N7H, 5C91
	MARCH8	Apoptosis, inflammation, and immune functions (Samji et al., 2014; Frost et al., 2019)	None reported.	2D8S
	RNF182	Neurotoxicity, reduced neurite length, and neuronal death (Liu et al., 2008; Mangieri et al., 2014).	None reported.	None reported.
Activated	Itch	Regulator of cell cycle re-entry that causes neural apoptosis. Immune pathway dysfunction and neuroinflammation (Venuprasad et al., 2015; Chauhan et al., 2020).	Clomipramine and norclomipramine (minimum inhibitory concentration of Itch autoubiquitination = 300 μM). Compound 10e (inhibits autoubiquitination at 5 μM) (Rossi et al., 2014; Liu et al., 2017).	5SXP, 5CQ2, 5C7M
	TRAF6	Inhibition of autophagy and resultant nerve injury as well as enhanced inflammation (Geetha et al., 2012; Dou et al., 2018; Vemula et al., 2020; Bandyopadhyay, 2021). Neuronal survival and spinogenesis (Geetha et al., 2012).	6877002 (141 μM), 6860766 (59 μM), C25-140 (2.8 μM) (Figure 3) (Zarzycka et al., 2015; Brenke et al., 2018).	1LB4, 1LB5, 3HCS, 3HCU, 3HCT
Downregulated	TTC3	Inhibition of neuronal differentiation, and control of the cell cycle (Berto et al., 2007; Suizu et al., 2009; Zhou et al., 2021).	None reported.	None reported.
	Ube3A	Synaptic function and plasticity (Sun et al., 2015; Owais et al., 2020).	OF227 (ubiquitination of RING1B between 50–100 μM following a 2 h incubation) (Figure 3) (Offensperger et al., 2020).	6U19, 1D5F, 1C4Z, 2KR1
	CHIP	Tau degradation and Aβ reduction (Petrucelli et al., 2004; Sahara et al., 2005; Singh and Pati, 2015).	CHIPOpt, YL-109 (increases CHIP transcription) (Figure 3) (Hiyoshi et al., 2014).	6EFK, 6NSV
	HRD1	ER associated degradation and promotion of APP ubiquitination and degradation to reduce Aβ (Kaneko et al., 2010; Shen et al., 2012; Zheng et al., 2016).	None reported.	5V6P, 5V7V
	Parkin	Mitochondrial turnover, synaptic deterioration, and neuronal death (Klein et al., 2006; Rodríguez-Navarro et al., 2007; Zhang C. W. et al., 2016; Quinn et al., 2020).	None reported.	4K95

#### NEDD4-1

While the HECT E3 ligase NEDD4-1 is ubiquitously expressed throughout all human tissues, its expression is notably high in the brain. Upregulation of NEDD4-1 has been noted in neurodegenerative diseases such as AD, Parkinson’s disease, amyotrophic lateral sclerosis, and Huntington’s disease (Kwak et al., 2012). Transcriptional activation due to an increase in neurotoxins or oxidative stress is reported to result in upregulation of NEDD4-1 (Kwak et al., 2012). Additionally, Kwak et al. (2012) demonstrated that a decrease in NEDD4-1 via shRNA or expressing a catalytically inactive form of NEDD4-1 in primary neurons rescues them from the neurotoxic effects of zinc-induced apoptosis. In brain lysates from AD patients and Aβ-treated neurons, elevated expression of Aβ causes localization and increased expression of NEDD4-1 in dendritic spines, leading to degradation and a resultant decrease in cell surface α-amino-3-hydroxy-5-methyl-4-isoxazolepropionic acid receptor (AMPAR) expression via endocytosis (Lin et al., 2011; Zhang et al., 2018). Loss of surface AMPARs is associated with synaptic changes in AD, including synapse weakening. Rodrigues et al. (2016) confirmed that NEDD4-1 is responsible for the detrimental Aβ-induced reduction of surface AMPARs, synaptic strength, and dendritic spine density in rat neurons. Furthermore, Kwak et al. (2012) reported increased expression of NEDD4-1 in frontal regions of AD patients and that likely contributes to synaptic dysfunction in AD (Rodrigues et al., 2016). These data suggest that the inhibition or downregulation of NEDD4-1 could improve synaptic plasticity and increase dendritic spine density in neurons. As further support cultured cortical neurons have increased NEDD4-1 expression after exposure to neurotoxins and upregulation of NEDD4-1 contributes to neuronal death (Kwak et al., 2012). Other essential roles of NEDD4-1 are related to terminal branching in retinal ganglion cells via downregulation of PTEN and increases expression of PI3K, and to neurite growth via Rap2 ubiquitination (Drinjakovic et al., 2010; Kawabe et al., 2010). These data support that increased NEDD4-1 expression and activity in AD exacerbates disease.

#### Itch (AIP4)

Itch is a HECT-containing E3 ubiquitin ligase that displays aberrant overactivation in AD. Chauhan et al. (2020) demonstrated that in cortical neurons derived from transgenic AD (TgAD) animals, activation of the c-Jun N-terminal kinase (JNK) pathway is induced by treatment with Aβ1–42 and results in hyperphosphorylation of Itch, which primes it for autoubiquitination and activation. Suppression of the neuronal cell cycle in terminally differentiated neurons is essential to avoid cell cycle re-entry that causes neuronal apoptosis (CRNA), a condition that plagues AD patients. As Itch acts as a major regulator of CRNA, eliciting its downregulation can reverse CRNA and reduce neuronal loss in AD (Chauhan et al., 2020). This represents an E3-mediated pathway that propagates neurodegeneration in AD. Another well-documented and essential role of Itch is in immune regulation. Via ubiquitinating key immune regulatory proteins, Itch regulates lymphocyte-cell activation and Itch deficiency is known to result in inflammatory disorders (Venuprasad et al., 2015). As immune dysfunction and neuroinflammation are prevalent in AD, it is possible that Itch overactivation also contributes to AD pathology via propagating aberrant immune pathways.

#### TRAF6

TRAF6 is an E3 ligase with a reported role in driving AD pathology due to its overactivation. TRAF6 activation in AD results in inhibition of autophagy and resultant nerve injury as well as enhanced inflammation (Dou et al., 2018). TRAF6-driven inflammation that ultimately leads to neuronal death in AD is mediated by NF-κB and MAPK signaling pathways, indicating that TRAF6 inhibition may be beneficial to AD patients. Specifically, microglia and astrocytes mediate inflammation when they are activated in response to Aβ interacting with toll-like receptor 4 (TLR4). This interaction leads to activation of MyD88 and TRAF6, which then stimulates NF-κB, AP-1, and MAPK signaling and results in release of pro-inflammatory cytokines (Bandyopadhyay, 2021). Pro-inflammatory cytokines such as IL-1β and TNFα and reactive oxygen species propagate neuronal damage in AD (Akama and Eldik, 2000; Dou et al., 2018; Bandyopadhyay, 2021). In a somewhat conflicting manner, TRAF6 plays roles in neuronal survival and activity, which include Aβ-neurotrophin receptor P75 (Aβ-p75^NTR^) pathways and spinogenesis, suggesting that inhibition of TRAF6 would be detrimental to AD patients (Geetha et al., 2012). Aβ binds to p75^NTR^ and inhibits polyubiquitination, leading to neuronal death. TRAF6/p62, however, can restore neuronal survival via abrogating the Aβ-mediated inhibition (Geetha et al., 2012). TRAF6 also controls early spinogenesis via binding to cell adhesion peptide neuroplastin and restores failed spinogenesis in neuroplastin-deficient neurons (Vemula et al., 2020). Vemula et al. (2020) demonstrated that blockage of TRAF6 function impairs the formation of excitatory synapses in rat hippocampal neurons. The seemingly disparate roles of TRAF6 in AD support the need for high-quality small molecules that modulate this E3 ligase and would allow for better characterization of its diverse functions in inflammation, neuronal viability, and spinogenesis.

#### RNF182

RNF182 is a RING finger containing transmembrane E3 ligase commonly expressed in neural tissues that has increased expression in post-mortem AD patient brains with evidence of neurodegeneration (Liu et al., 2008; Kaneko et al., 2016; Okamoto et al., 2020). Liu et al. (2008) demonstrated that upon subjecting NT2 neurons, which are derived from a human tetracarcinoma, to oxygen and glucose deprivation (OGD) that RNF182 expression was increased, and neuronal cell death occurred. As a potential mechanism to explain this finding, RNF182 elicits the degradation of ATP6V0C, an integral protein for gap junctions and neurotransmitter release channels (Liu et al., 2008). ATP6V0C knockdown in differentiated SH-SY5Y neuroblastoma cells induced markers of neurotoxicity and reduced neurite length, further supporting the proposed mechanism by which RNF182 overexpression results in neuronal death in AD and that its inactivation, inhibition, or degradation could be of benefit to AD patients (Mangieri et al., 2014).

#### MARCH8 (c-MIR or RNF178)

MARCH8 plays a key role in the immune response, partially through regulation of cytokines such as TNF-α and IL-6 (Samji et al., 2014). An increase in TNF-α levels can trigger neurotoxicity in AD and other neurodegenerative diseases. After stimulation of cultured neurons with TNF-α, the mRNA and protein expression levels of MARCH8 are increased (Guo et al., 2019). MARCH8 co-localizes with myosin light chain 2 (MLC2) in hippocampal neurons and is proposed to be responsible for the degradation of MLC2. Furthermore, the MARCH8-MLC2 interaction is increased in response to TNF-α treatment. Guo et al. (2019) demonstrated that siRNA specific to MARCH8 decreases apoptosis following TNF-α treatment (Frost et al., 2019). Overexpression of MARCH8 in AD can result in downregulation of several immunomodulatory receptors, impaired development of immune cells, and resultant immune suppression (Samji et al., 2014). This role of MARCH8 in inflammation and immunity, in addition to regulation of apoptosis, could propagate AD pathology.

### Downregulated or Less Active E3 Ligases

Decreased expression and/or reduced activity of certain E3 ligases has been observed in AD patients (Figure 2 and Table 1). E3 ligases that are reported as downregulated in AD include carboxy-terminus of Hsc70 interacting protein (CHIP), tetratricopeptide repeat protein 3 (TTC3), Ube3A, HRD1, and Parkin. Either rescuing or enhancing the activity of these E3 ligases where mutation and/or reduced expression has resulted in loss-of-function may be beneficial for AD patients.

#### TTC3

TTC3 is an E3 ligase associated with neuronal differentiation. Some AD patients have a rare TTC3 mutation that causes downregulation of the associated protein (Hu et al., 2020; Schmidt et al., 2021). Kohli et al. (2016) determined that in 11 late-onset AD (LOAD) individuals there was a missense mutation in TTC3 from a single variant, rs377155188 (p.S1038C), with a serine to cysteine switch. The source and consequences of this rare mutation are still being characterized. This motivated Laverde-Paz et al. (2021) to report the generation of human induced pluripotent stem cell lines derived from a neurologically normal male that contain the homozygous TTC3 mutation to help elucidate the mechanisms surrounding this mutation. Additionally, TTC3 protein expression is reduced in all LOAD patients (Kohli et al., 2016). Although TTC3 was found to increase susceptibility to LOAD, its expression has not been associated with initiating AD pathogenesis (Zhou et al., 2021). TTC3 is reported to have a role in ubiquitination and degradation of phosphorylated AKT, inhibition of neuronal differentiation, and control of the cell cycle (Berto et al., 2007; Suizu et al., 2009). These essential roles and loss of TTC3 function in AD support that mechanisms that restore TTC3 activity could benefit AD patients.

#### CHIP

The CHIP contains three tetratricopeptide repeats (TPRs) and is a U-box-containing E3 ubiquitin ligase with roles in tauopathy and amyloidopathy in AD (Joshi et al., 2016). CHIP is a brain enriched E3 ligase and a decrease in the expression of CHIP in transgenic mice and cultured cells has been associated with Aβ accumulation (Singh and Pati, 2015). Sahara et al. (2005) reported that decreased levels of CHIP have been observed in AD patient brains that have elevated paired helical filaments-tau (PHF-tau) and CHIP levels correlate with Hsp90 levels. Additionally, JNPL3 mice with P301L tau overexpression exhibit decreased CHIP levels in their spinal cords, increased tau inclusions, and neuronal loss, whereas their cerebellar regions demonstrated increased CHIP expression (Sahara et al., 2005). Zhang et al. (2020) showed that CHIP can directly bind, attach K48- and K63-linked ubiquitin, and eliminate phosphorylated tau, inducing both proteasomal and autophagic degradation (Joshi et al., 2016). CHIP also interacts with caspase-cleaved tau and is involved in its degradation in cortical neurons (Dolan and Johnson, 2010). Complexes between heat shock proteins (HSPs) and CHIP can increase the degradation of Aβ42 peptide and provide resultant protection against Aβ toxicity in neurons (Kumar et al., 2007). Alternatively, Singh and Pati (2015) demonstrated that in rat cortical neurons, CHIP ubiquitinates beta-secretase 1 (BACE1) and induces its proteasomal degradation, which reduces APP processing, stabilizes APP, and reduces Aβ levels. Additionally, in HEK-APP cells CHIP stabilizes p53 and causes transcriptional repression of BACE-1, providing another mechanism for APP stabilization (Singh and Pati, 2015). These data indicate that overexpression or activation of CHIP could be beneficial in AD and related tauopathies.

#### Ube3A (E6AP)

The HECT E3 ligase Ube3A has described roles in synaptic function and plasticity, both of which are affected in AD patients (Sun et al., 2015). Synaptic dysfunction and synapse loss are prominent features of AD (Olabarria et al., 2019). In a mouse model that overexpresses APPswe, resulting in accumulation of Aβ in the brain, Ube3A expression is reduced (Olabarria et al., 2019). In an APPswe/PS1ΔE9 double mutant transgenic mouse model, expression of Ube3A was reduced as the animal aged (Singh et al., 2017). Furthermore, Singh et al. (2017) reported that in maternal Ube3A-deficient mice levels of peroxisome proliferator-activated receptor-α (PPAR-α) were significantly increased and resulted in ADAM10 upregulation. ADAM10 overexpression increased α-secretase cleavage of the N-terminal fragment of APP (sAPPα) and decreased Aβ levels. Ube3A-deficient mice also exhibited cognitive and motor deficits as well as an increased propensity toward obesity (Singh et al., 2017). Finally, the estrogen receptor (ESR2/ER-β), which has roles in long-term potentiation and synaptic strength, is a substrate of Ube3A. Ube3A activates transcription of the *ESR2* gene, which encodes ER-β. Overexpression of ESR2 in a rat model of AD reduces Aβ hippocampal deposits and improves the learning and memory of AD rats, solidifying its role in the development and progression of AD and a desire to target it for either inhibition or degradation (Owais et al., 2020).

#### HRD1 (SYVN1)

HRD1 is an E3 ubiquitin ligase expressed in the ER membrane of brain neurons responsible for endoplasmic reticulum-associated degradation (ERAD) (Kaneko et al., 2010; Saito et al., 2014; Zheng et al., 2016). HRD1 colocalizes with APP and regulates its levels via ubiquitination and degradation in brain neurons, resulting in decreased generation of Aβ (Kaneko et al., 2010; Anuppalle et al., 2013). Kaneko et al. (2010) reported decreased protein levels of HRD1 in the cerebral cortex of AD patient brains (Zheng et al., 2016; Schmidt et al., 2021). Oxidative stress in AD is suggested to affect HRD1 solubility and lead to its accumulation in the aggresome, which is an aggregation of misfolded proteins formed when the protein degradation system is overwhelmed or dysfunctional (Chin et al., 2008; Saito et al., 2014). It has been suggested that suppression of HRD1-mediated ERAD leads to Aβ generation, ER/oxidative stress and apoptosis, all of which could propagate AD pathology (Kaneko et al., 2010). Finally, an inverse relationship between HRD1 and p-tau accumulation in hippocampal neurons in AD support that HRD1 may be a negative regulator of p-tau (Shen et al., 2012). Reduced expression of HRD1 therefore promotes tau cytotoxicity and reduces cell survival in AD.

#### Parkin

Parkin is an RBR E3 ubiquitin ligase involved in mitochondrial physiology that is more commonly associated with Parkinson’s disease but plays a role in pathogenesis of AD and other neurodegenerative diseases (Quinn et al., 2020). Essential proteins in mitochondrial autophagy, LC3 and p62, are recruited alongside Parkin to damaged mitochondria in neurons from mutant hAPP-expressing transgenic mice and AD patient brains (Chakravorty et al., 2019). Parkin expression is downregulated in AD patient brains, resulting in dysfunctional mitophagy (Zheng et al., 2016; Quinn et al., 2020). Ye et al. (2015) demonstrated that AD neurons have increased Parkin recruitment to depolarized mitochondria, suggesting that AD-related mitochondrial stress can induce Parkin-mediated mitophagy (Chakravorty et al., 2019). Furthermore, Klein et al. (2006) reported that overexpression of Parkin in a tau neurodegenerative model protects dopaminergic (DA) neurons against toxicity (Rodríguez-Navarro et al., 2007). Finally, Corsetti et al. (2015) showed that an N-terminal truncated tau in AD patients can induce deregulated mitophagy via recruitment of Parkin, resulting in excessive mitochondrial turnover, synaptic deterioration, and neuronal death. Parkin deficiency is suggested to precipitate tau pathology and amyloid burden, and deter hippocampal long-term potentiation, thus exacerbating disease for AD patients (Zhang C. W. et al., 2016).

## E3 Ligases Implicated in Alzheimer’s Disease With Published Structural Data and/or Ligands

### Types of Structures Solved

**FIGURE 3:**
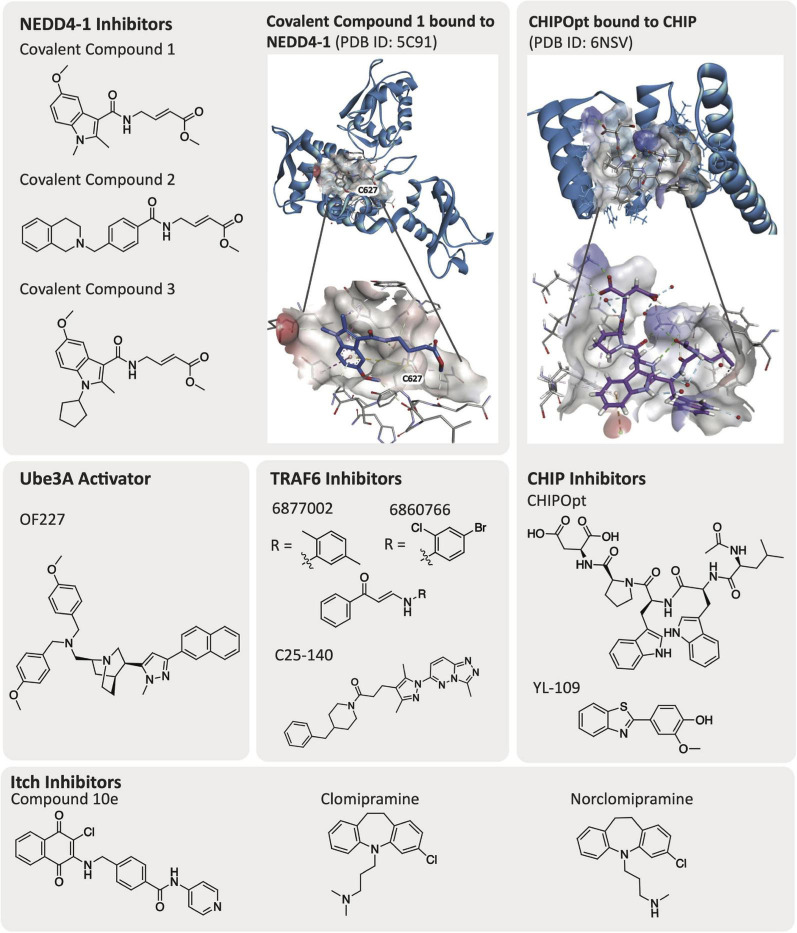
Published E3 ligase modulators and solved ligand-bound co-crystal structures.

Elucidation of the structure of E3 ligases via crystallographic or NMR-based methods greatly facilitate drug discovery efforts. Currently, a number of E3 ligases that have been linked to AD pathology, several of which were described in the previous section, have been crystallized and have solved structures: CHIP, NEDD4-1, TRAF6, Parkin, HRD1, Ube3A (E6AP), Itch, and MARCH8 (Table 1). Additionally, co-crystallographic structures of a few E3 ligases with peptides or small molecule ligands bound have been disclosed. The availability of co-crystal structures is pivotal and creates the potential for more expedient ligand design. While not essential for small molecule inhibitor development, an available structure enables computational studies and structure-based drug design as integral parts of the ligand development process. CHIP has been co-crystallized with its corresponding native peptides and an optimized peptide ligand (Figure 3). NEDD4-1 is unique as it has published co-crystal structures with both peptides and covalent small molecule inhibitors (Figure 3). To our knowledge, there is no crystallographic data for E3 ligases TTC3 and RNF182. We highlight a few of these structures and what has been learned through their public availability in the section below.

### Specific Structures Solved and Mechanistic Relevance

#### CHIP

The CHIP E3 ligase consists of three TPRs and a U-box domain (McDonough and Patterson, 2003; Wang T. et al., 2020). The TPR domains of CHIP E3 ligase bind to the C-terminus of chaperone proteins such as Hsp70/90, while the CHIP U-box domain is responsible for its E3 ubiquitin ligase activity (Zhang et al., 2005, 2020). The TPR domains are also reported to bind directly to tau^*D*421^ and caspase6^*D*179^, supporting the observation that loss of CHIP increases accumulation of tau^*D*421^ (Ravalin et al., 2019). Posttranscriptional loss of CHIP was shown to coincide with increased levels of tau^*D*421^ and caspase6^*D*179^ in hippocampal tissue from Braak VI and Braak III AD patients (Ravalin et al., 2019). Direct binding of the CHIP TPR domain to tau^*D*421^ was also confirmed experimentally via a fluorescence polarization (FP) assay and mutation of CHIP^*WT*^ to CHIP^*K*30*A*^, which is a mutation in the carboxylate clamp, as it resulted in decreased ubiquitination of tau^*D*421^ and further confirmed the interaction with the TPR. The CHIP TPR domain is co-crystallized with 5-mer acetylated peptides from Hsp70 [Ac-IEEVD; Protein Data Bank (PDB) ID: 6EFK] and an optimized CHIP binding peptide sequence named CHIPOpt (Ac-LWWPD; PDB ID: 6NSV, Figure 3), providing structural rationale for the increased binding affinity observed with respect to CHIPOpt (Ravalin et al., 2019).

#### NEDD4-1

NEDD4-1 consists of a HECT domain that contains two cysteine residues important for ubiquitylation events and a catalytic C2 domain that are linked by a region with three WW domains (Mari et al., 2014). WW domains are constructed of peptide chains folded into a triple stranded β-sheet that contain two conserved tryptophan (W) residues (Ingham et al., 2004). NEDD4-1 remains in an auto-inhibited state where the C2 domain interacts with the HECT domain until it is activated by a substrate binding to the WW domain. An E2 ligase transfers a ubiquitin to Cys867 on the HECT domain C-lobe, which is then transthiolated to the HECT domain N-lobe Cys627 before finally being transferred to the substrate and resulting in its proteasomal degradation (Kathman et al., 2015). There are solved crystal structures of the HECT, WW, and ubiquitylated HECT domains available in the PDB. In particular, the co-crystal structures of PPXY peptide motifs bound to the WW domain of NEDD4-1 (PDB ID: 4N7H) and the HECT domain with a small molecule covalently bound to Cys627 (PDB ID: 5C91, Figure 3) are beneficial for developing additional compounds targeting NEDD4-1 (Kathman et al., 2015; Qi et al., 2014).

#### Itch

Itch has a HECT domain, four WW domains, and a proline rich domain (PRR) between the WW and C2 domain (Shah and Kumar, 2021). Similar to NEDD4-1, Itch remains in an autoinhibited state with its WW domains bound to its HECT domain and must undergo a conformational change to reveal its catalytic cysteine to accept ubiquitin (Riling et al., 2015). Itch interacts with PPXY motifs via its WW domains and can interact with Src homology 3 (SH3) domains via its PRR, ubiquitinating proteins that bear these motifs (Desrochers et al., 2017). Desrochers et al. (2017) crystallized the Itch PRR with the β-PIX SH3 complex (PDB ID: 5SXP) and described “super-SH3” domains, which they defined as one PRR engaging two SH3 domains. The authors rationalize this complex interaction as evidence to support a lack of β-PIX ubiquitination by Itch. Structural data of Itch WW3 and WW4 domains bound to a peptide (PPCY peptide) from a substrate of Itch, thioredoxin-interacting protein (TXNIP), highlights key binding regions in these domains (PDB ID: 5CQ2) (Liu et al., 2016). As experimental support of these structural findings, Zhang C. W. et al. (2016) generated several ubiquitin variants (UbVs) for HECT E3 ligases to enable modulation of their function via inhibition or activation. UbVs take advantage of the large, solvent-accessible surface of ubiquitin mediates low-affinity interactions with a variety of proteins. This surface is amenable to engineering to diversify it, resulting in optimized UbVs that bind tightly and with high selectivity to a particular ubiquitin-interacting protein (Teyra et al., 2019). In general, it has been found that a UbV that binds to the E2-binding site inhibits E3 ligase function, while a UbV that occupies the ubiquitin-binding exosite results in activation of E3 ligase function. The UbV IT.2 displayed an inhibitory effect on Itch, disrupting the E2 ligase binding region rather than impeding ubiquitin transfer, which was confirmed by crystallography of UbV IT.2 with Itch (PDB ID: 5C7M) (Zhang C. W. et al., 2016). UbV IT.2 hydrophobic residues interact with the canonical binding sites of the E3 ligase Ubch7. Characterization of this inhibitory interaction provides key structural data that could be used to identify novel small molecule ligands for Itch.

#### Parkin

The structure of Parkin consists of an N-terminal ubiquitin-like domain (Ubl) and four RING-like domains (RING0, RING1, IBR, and RING2) (Seirafi et al., 2015). Individual crystal structures of these RING domains have been solved with good resolution that are available in the PDB. Additionally, the full-length protein was crystallized (PDB ID: 4K95), albeit at a lower resolution of 6.5 Å (Seirafi et al., 2015). The full-length crystal structure provided insight into the activation of Parkin, and elucidated that Parkin exists in an autoinhibited state (Trempe et al., 2013). Based on crystallographic data, Sauvé et al. (2018) hypothesize that Parkin binds to phosphorylated-Ub, generated by PINK1, decreasing the binding of RING1 to the Ubl domain and leading to PINK-mediated phosphorylation of the dissociated Ubl domain. This phosphorylation event releases the Ubl domain to bind to RING0, which subsequently releases the RING2 domain and the repressor element of Parkin (REP) linker due to steric clashes. The E2 binding site of Parkin can interact with ubiquitin and a specific E2 enzyme (Sauvé et al., 2018). The mechanistic insights revealed through structural biology offer benefit in the development of novel chemical entities that could potentially activate Parkin and alleviate the mitochondrial dysfunction caused by reduced Parkin levels in AD (Quinn et al., 2020).

#### MARCH8

The structure of MARCH8 consists of a RING-CH domain, two transmembrane domains, two tyrosine-based motifs, and a PDZ domain, named for a commonly shared structural domain consisting of six β-sheets and two α-helices (Romero et al., 2011; Lin et al., 2019). Fujita et al. (2013) reported that the membrane-associated RING-CH domain is responsible for ubiquitination of substrates such as transferrin receptor, leading to its lysosomal degradation. Similar to other RING-type ubiquitin ligases, this RING-CH domain is located in the N-terminal cytoplasmic domain. A six-amino-acid sequence located in the C-terminal domain of MARCH8, which is highly conserved among different species, is required for substrate recognition and ubiquitination (Fujita et al., 2013). A solution structure of the RING-CH domain (PDB ID: 2D8S) was reported, however, there has been no further crystallography reported of MARCH8 domains or full structures. As MARCH8 is suggested to form homo- and heterodimers with other MARCH family E3 ligases via their transmembrane domains, many interesting structures and points of chemical intervention could be envisioned with respect to this AD-relevant E3 ligase (Samji et al., 2014).

#### TRAF6

TRAF6 consists of a RING domain, five zinc finger domains, a TRAF-N domain, and a TRAF-C domain. There is high structural homology between TRAF6 and TRAF2, TRAF3, and TRAF5 (Park, 2018). Ye et al. (2002) resolved the crystal structures of the TRAF6 TRAF-C domain apo structure (PDB ID: 1LB4) as well as the TRAF-C domain bound to either a CD40 (PDB ID: 1LB6) or RANK peptide (PDB ID: 1LB5). As the TRAF-C domain of TRAF6 binds different peptide motifs versus the homologous TRAF2, TRAF3, and TRAF5, structural elucidation could be helpful in achieving selectivity within the family. Mercier et al. (2007) solved a solution structure of the TRAF6 RING domain via high resolution ^1^H, ^13^C, and ^15^N-NMR spectroscopy. Yin et al. (2009) published the structure of the N-terminal domain of TRAF6 (PDB ID: 3HCS). This group also crystallized the TRAF6 RING domain and three zinc fingers with E2 ubiquitin ligase Ubc13, revealing that TRAF6 interacts with Ubc13 via its RING finger and that its first zinc finger plays an important structural role (PDB ID: 3HCU, 3HCT). Finally, Middleton et al. (2017) crystallized a TRAF6 dimer with a Ubc13-Ub conjugate and demonstrated that the first zinc finger is important for stabilizing ubiquitin and aids in deposition of K63-linked chains. Mutagenesis studies experimentally confirmed that dimerization of TRAF6 is important for polyubiquitin synthesis and autoubiquitination.

#### HRD1

HRD1 E3 ligase is a membrane protein consisting of a signal peptide, transmembrane domain, RING finger domain and a proline-rich domain (Omura et al., 2008). HRD1 reportedly forms a complex with three membrane proteins (HRD3, USA1, and DER1) and a luminal protein (YOS9), and HRD3 and YOS9 are responsible for regulating HRD1 E3 ligase activity (Schoebel et al., 2017). Schoebel et al. (2017) reported the cryo-EM structures of HRD1-HRD3 complexes with one or two HRD3 molecules bound, respectively (EMD-8639, PDB ID: 5V6P and EMD-8638, PDB ID: 5V7V). Wu et al. (2020) recently used cryo-EM analysis to characterize the structure of the active HRD1 complex, which consists of HRD1, HRD3, DER1, USA1, and YOS9 proteins. In mouse neurons, APP binds to HRD1 at proline rich regions, leading to the ubiquitination and degradation of APP (Nomura et al., 2016).

#### Ube3A

Ube3A is a HECT E3 ligase that has an amino-terminal zinc-binding (AZUL) domain, HERC2 binding domain, E6-binding domain and a HECT domain (Owais et al., 2020). The AZUL domain is unique to this E3 ligase, and it mediates the interaction between Ube3A and a Ube3A-binding domain (RAZUL) in the proteasome substrate receptor hRpn10 (RAZUL:AZUL PDB ID: 6U19). Characterization of this site and specific interaction confirms that Ube3A ubiquitinates substrates at the proteasome (Buel et al., 2020). Additional crystal structures are resolved for the HECT catalytic domains either alone (PDB ID: 1D5F) or with a partner protein (UBCH7, PDB ID: 1C4Z) and a solution structure was solved for the zinc- binding N-terminal domain (PDB ID: 2KR1) (Lemak et al., 2011; French et al., 2017). The amount of structural work around this E3 ligase should aid in the design of additional Ube3A ligands.

### Published Ligands for Alzheimer’s Disease-Relevant E3 Ligases

**FIGURE 4:**
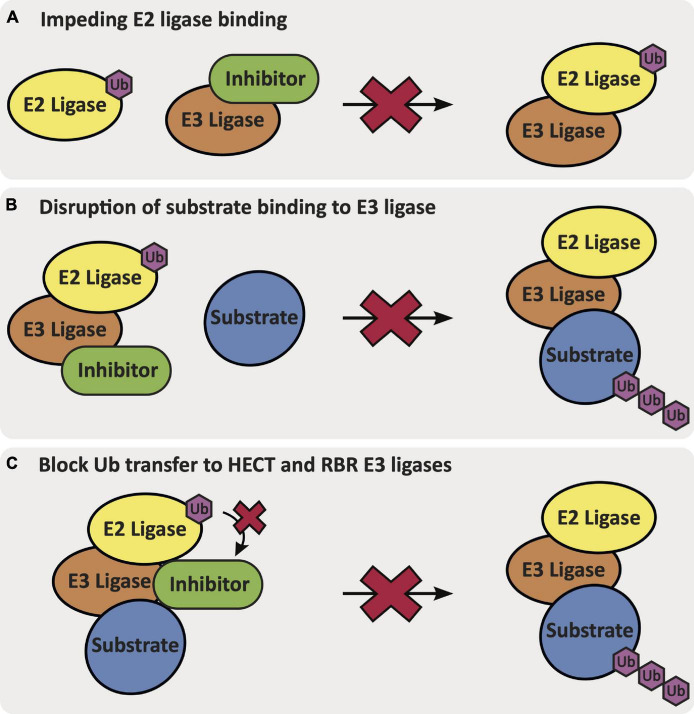
The mechanisms via which an inhibitor binds and inhibits E3 ligase function: **(A)** impeding E2 ligase binding to the E3 ligase, **(B)** disruption of native substrate binding to the E3 ligase, or **(C)** blocking Ub transfer from the E2 ligase onto the HECT or RBR E3 ligase.

Ligands that bind to specific E3 ligases in a selective manner will be impactful in elucidating mechanisms and the therapeutic potential of these putative targets within AD. Ligands that can activate the function of an E3 ligase that is downregulated and/or has loss-of-function mutations could aid restoration of biological function(s) that may slow AD progression. Alternatively, E3 ligases can be inhibited via different mechanisms: (1) by impeding E2 ligase binding to the E3 ligase, (2) by disrupting substrate binding of the E3 ligase, or (3) for HECT and RBR E3 ligases that require an intermediatory transthiolation of ubiquitin, by blocking the transfer of ubiquitin onto the E3 ligase (Figure 4) (Spratt et al., 2014; Weber et al., 2019). E3 ligands can be either small molecules or peptides, and examples of each have been described. The development of small molecule ligands that have favorable physicochemical and pharmacokinetic/pharmacodynamic properties is desirable, and ultimately optimization of a ligand for blood–brain barrier penetration is necessary for AD-related targets in the brain. Peptidic ligands are often designed based on the shortest peptide sequence found within the binding domain of endogenous binding partners. By nature, these peptidic ligands are not drug-like but can provide valuable starting points for small molecule ligand development. One final category of E3 ligase-binding ligands includes bifunctional molecules, such as PROTACs and deubiquitinase-targeting chimeras (DUBTACs), which bring E3 ligases in proximity with another protein to promote either degradation or stabilization. The development of PROTACs that can induce degradation of previously undruggable targets using either small molecule or peptide ligands has provided an alternative approach to modulation of protein function (Paiva and Crews, 2019). PROTACs demonstrate different properties than small molecule inhibitors and act via an event-driven pharmacology as opposed to an occupancy-driven pharmacology (Lai and Crews, 2017). Unique characteristics of the event-driven pharmacology elicited by PROTACs includes their catalytic activity and ability to remove function until a protein is resynthesized. Additionally, the specificity of degradation of the protein of interest (POI) is not governed by the specificity of the parent ligand, but by the ternary complex interface formed between the E3-PROTAC-POI (Lai and Crews, 2017; Smith et al., 2019). Recently, the development of DUBTACs has furnished bifunctional molecules that are capable of stabilizing proteins. DUBTACs have utility in diseases like AD when the ubiquitination and degradation of key proteins drives disease pathology. These compounds provide a way to stabilize the actively degraded target and therein preserve its essential function, slowing disease progression (Henning et al., 2021). Examples of known ligands for AD-relevant E3 ligases are included in the following subsections (Table 1). Although the ligands listed below are beneficial for beginning to understand the effects of modulating the function of these E3 ligases, it is important to note that none of these ligands fit in the criteria to be considered a chemical probe, highlighting the need for additional research effort. A chemical probe has sufficient on-target potency, selectivity, a defined mechanism of action, full characterization to enable *in vitro* use to address a specific biological hypothesis, and must be widely available to the scientific community (Frye, 2010).

#### NEDD4-1

Low potency inhibitors of NEDD4 subfamily member NEDD4-1 exist, including inhibitors of the WW domains and covalent inhibitors that bind the cysteine residues in either the N-lobe (Cys627) or C-lobe (Cys867). Current inhibitors of the WW domains of HECT E3 ligases are phospho (pY)-containing peptides that mimic endogenous substrate binding with low micromolar potency (Harvey et al., 2002; Kanelis et al., 2006; Panwalkar et al., 2016; Zhu et al., 2017). Kathman et al. (2015) developed small molecule covalent inhibitors of NEDD4-1 (covalent compounds 1–3, Figure 3) that specifically bind at Cys627 on the N-lobe of NEDD4-1 selectively over Cys867 in the C-lobe, despite Cys867 being more reactive (Rossi et al., 2014; Chen et al., 2018). Interestingly, Kathman et al. (2015) observe that the covalent inhibitors do not entirely impede ubiquitination of substrates, but rather change the mechanism of ubiquitination from processive to distributive. The most potent of these inhibitors was covalent compound 3, which was able to disrupt the binding of NEDD4-1:Ub (covalent compound 3, *K*_*I*_ = 29.3 μM, Figure 3) and prevent NEDD4-1 from forming long polyubiquitin chains on a substrate (Wbp2-C-K222) due to a distributive mechanism of ubiquitination (Kathman et al., 2015).

#### Ube3A

Small molecule activators that rescue the E3 ligase activity of wild-type as well as Angelman syndrome variants of Ube3A/E6AP have been identified (Offensperger et al., 2020). Expression of E6AP can be altered either prior to or following its translation. Offensperger et al. (2020) discovered through a small molecule high-throughput FP screen that certain small molecule compounds could act as E6AP activators that stabilize the E3 ligase in an enzymatically active state. A screen of 48,000 compounds yielded an initial set of seven structurally similar compounds that could activate E6AP. Four of these compounds, OF204, OF227, OF232, and OF234, elicited E6AP autoubiquitination as well as ubiquitination of RING1B. It was noted that of the original hits, OF204, OF232, and OF234 interfered with E1 activity, however, at higher concentrations than required for E6AP activation. OF227 (Figure 3) was identified as the best activator of E6AP and was found to elicit ubiquitination of RING1B between 50–100 μM following a 2 h incubation.

#### CHIP

Small molecules that can induce CHIP expression have been described (Zhang et al., 2020). Although a study of the broader cellular effects that these molecules elicit has not been executed, small molecules have been described that increase the expression of CHIP E3 ligase. For example, the small molecule YL-109 (Figure 3) was shown to increase CHIP transcription in breast cancer cells (MDA-MB-231) via recruitment of aryl hydrocarbon receptor (AhR) upstream of the CHIP gene (Hiyoshi et al., 2014).

#### Itch

Small molecules developed to inhibit Itch activity are focused on blocking Itch autoubiquitination. In a high-throughput enzyme-linked immunosorbent assay (ELISA) screen, Rossi et al. (2014) discovered that clomipramine and an active metabolite, norclomipramine, inhibited Itch autoubiquitination. Potential off-target binding to the E1 or E2 ligase was ruled out for both of these compounds and an *in vitro* minimum inhibitory concentration value of 300 μM was determined with respect to inhibition of Itch autoubiquitination. A more recent study by Liu et al. (2017) investigated 1,4-naphthoquinones as Itch inhibitors, leading to the identification of compound 10e as an *in vitro* inhibitor of Itch ubiquitination at 5 μM. Intriguingly, the inhibitor also decreased Itch protein levels via western blot analysis at 10 μM in a human RPMI8226 multiple myeloma cancer cell line. The mechanism of action of the inhibitor has not yet been elucidated and there was not any evidence of direct binding to Itch presented (Liu et al., 2017).

#### TRAF6

In targeting TRAF6, investigators have pursued the identification of small molecule protein–protein interaction (PPI) disruptors. A small molecule inhibitor of TRAF6, C25-140 (Figure 3), was developed by Brenke et al. (2018) that inhibits TRAF6 activity via disruption of its interaction with Ubc13 (IC_50_ = 2.8 μM). The inhibitor was screened for selectivity against other E3 ligases and was shown to be selective for TRAF6 versus HECT, SUMO, and RING E3 ligases such as MDM2, TRIM63, Itch, E6AP, and RNF4. In addition, C25-140 did not act as an E1-E2 PPI disruptor. However, C25-140 did inhibit cIAP1, which, like TRAF6, attaches K63-linked ubiquitin chains to its substrates. Inhibition of TRAF6-Ubc13 with C25-140 decreased NF-κB activation in primary human peripheral blood mononuclear cells (PBMCs) and in *ex vivo* primary murine T cells. Another small molecule that acts as a PPI disruptor of TRAF6-CD40 is 6877002 (Figure 3), which was identified via a combination of *in silico*, *in vitro*, and *in vivo* experiments. When analyzed via surface plasmon resonance (SPR), 6877002 was found to also weakly bind to the C-terminal domains of TRAF1, TRAF2, TRAF3, and TRAF6 with binding affinities of 142, 144, 99, and 141 μM, respectively (Zarzycka et al., 2015). This compound is reported to reduce CD40-induced TNF production and increase expression of the anti-inflammatory cytokine IL-10 in human monocytes. Overall, inhibition of the CD40-TRAF6 interaction with 6877002 impairs the recruitment of monocytes and macrophages to the CNS and reduces neuroinflammation (Aarts et al., 2017). A structurally similar small molecule inhibitor to 6877002, 6860766, also binds to the C-terminal domains of TRAF1, TRAF2, TRAF3, and TRAF6 with binding affinities of 51, 30, 37, and 59 μM, respectively (Zarzycka et al., 2015).

## Screening Approaches to Identify New E3 Ligase Modulators

Approaches to identify novel modulators of E3 ligase activity that can either inactivate or activate the E3 ligase are instrumental to enhance understanding of E3 ligase biology in AD. In addition to identifying compounds that bind to the E3 ligase, understanding the endogenous substrates of the E3 ligase can also be a fundamental tool for understanding disease propagating pathways. Only through these screening approaches will the field be able to expand the repertoire of available E3 ligase ligands and enable investigation of biology mediated by these interesting proteins in relation to AD.

### Inhibition of Ubiquitin-Mediated Transfer of E3 Ligases

Multiple assays have been developed to identify ways to inhibit E3 ligases that are overactive or overexpressed in AD. The function of E3 ligases can be inhibited via impeding ubiquitin transfer from the E2 ligase to the E3 ligase or inhibiting the E2 ligase from interacting with its E3 ligase (Figure 4). Direct inhibition of E3 ligase activity can be achieved via targeting and/or modifying the cysteine responsible for transthiolation of ubiquitin in the case of RBR and HECT family E3 ligases (Figure 4). Inhibition of E3 ligase function can be determined via mass spectrometry (MS), luminescence- and/or fluorescence-based methods.

The activity of E2 and E3 ligases can be determined via measuring the depletion of mono-ubiquitin using a high-throughput matrix-assisted laser desorption/ionization-time of flight (MALDI-TOF) assay (Cesare et al., 2018). An assay of this type is built on knowledge that in the absence of a substrate, an E3 ligase will either produce polyubiquitin chains or undergo autoubiquitylation, which can be monitored via ^15^N-labeled ubiquitin internal standards to observe the decrease in monoubiquitin via MALDI-TOF MS (Cesare et al., 2018). This method facilitates identification of specific E2/E3 substrate pairings that can be further used to develop a high-throughput assay specific to an E2/E3 ligase pair in pursuit of an inhibitor of their activity. Cesare et al. (2018) screened for inhibitors of MDM2 (a RING E3 ligase), Itch (a HECT E3 ligase), and HOIP (RNF31, a RBR E3 ligase) using a library of 1,430 compounds. Although this assay does not distinguish between inhibitors of E1/E2/E3 ligases, it pinpoints loss of activity of these pairings. MALDI-TOF MS can then be used to delineate specific interactions between the inhibitor and the individual E1/E2/E3 ligases. Cesare et al. (2018) were able to identify inhibitors of the E1-E2-Itch complex, however, they were found to be inhibitors of E2 ligase activity. Strategies to block E3 ligase activity by targeting cysteine residues responsible for ubiquitin transthiolation on RBR and HECT E3 ligases have been used to identify covalent cysteine modifiers (Johansson et al., 2019).

Electrochemiluminescence-based assays that require activation of the E3 ligase with E1 and E2 ligases plus ATP can be utilized for high-throughput screening to identify E3 ligase inhibitors (Kenten et al., 2005). This methodology relies on adhering the GST-E3 ligase to a plate coated with glutathione electrodes, followed by addition of an E1-E2-Ub mixture and putative inhibitors. The anti-Ub antibody with an appended MSD sulfa-TAG binds to ubiquitin and enables detection of ubiquitination via electrochemiluminescence. This method was employed in a screen of 10,000 compounds in an attempt to identify an inhibitor of the E3 ligase RNF28.

A ubiquitin ligase profiling (ULP) system was developed that utilizes a high-throughput cellular luciferase reporter assay format to evaluate the inhibition of RING type E3 ligases (Maculins et al., 2016). The assay relies on a Gal4-Firefly luciferase (FL) reporter assay with a Gal4 DNA-binding domain fused to the E3 ligase of interest. Upon ubiquitination of the E3 ligase, Tandem Ubiquitin Affinity Entities (TUBEs) can bind to ubiquitin, leading to recruitment of a fused VP16 activation domain (ACT) to the minimal TATA box of the Gal4-firefly luciferase and a resultant luciferase signal. The ULP system was applied to screen for inhibitors of the E3 ligase RNF8 with a secondary screen for selectivity against TRAF6.

### Dual Inhibition/Activation Assays

Due to a need to regulate E3 ligases in AD, either via inhibition or activation, assays that have a dual readout and can be used to identify either inhibitors or activators in a high-throughput format are very useful. Examples of these types of assays are described below.

Homologous to E6AP C-terminus and RBR E3 ligases require transthiolation of a ubiquitin onto a catalytic cysteine residue prior to transfer of ubiquitin to the substrate. HECT E3 ligases have the catalytic cysteine in the HECT domain on their C-terminus, while RBR E3 ligases have the cysteine residue present in the RING2 domain, which also conjugates to ubiquitin (Smit and Sixma, 2014; Weber et al., 2019). Traditionally assays aimed at probing E3 ligase activity require an E1 ligase, E2 ligase, and ATP. The development of probes attached to ubiquitin, however, allow efficient transfer of ubiquitin to the E3 ligase via transthiolation followed by ligation to a substrate without the need for these components (Park et al., 2017). This system that does not require E1 and E2 ligases or ATP is called a ‘bypassing system’ (ByS) (Park et al., 2014).

The first example of one of these probes, called a ubiquitin mercaptoethanesulfonate (UbMES), is a ubiquitin C-terminal thioester probe that contains a mercaptoethanesulfonate group (Park et al., 2014, 2017). The effectiveness of the interaction of the E3 ligase catalytic cysteine with the UbMES probe is analyzed via western blot analysis, probing the extent of ubiquitination of the substrate, and allowing qualitative visual detection of inhibition or activation of substrate ubiquitination. A fluorescent labeled cysteine (FCys) can be added to quantitatively assess the enzyme kinetics of the HECT E3 ligase in this system. Park et al. (2014, 2017) demonstrated that other E3 ligases can adopt the ByS, including HECT E3 ligases (NEDD4-1, NEDD4-2, WWP1, and Itch) and RBR E3 ligases (Parkin and HHARI). A similar assay was developed called Ubfluor that releases a FP signal after ubiquitin is transferred to the E3 ligase, and inhibition of this transfer via introduction of covalent inhibitors quenches the signal (Krist et al., 2016; Foote et al., 2017). This FP format allows for a more high-throughput and quantitative assay that is not reliant on western blot analysis or addition of FCys and can be used to directly screen for inhibition of the catalytic cysteine in real time.

Tian et al. (2019) used a ubiquitin reference technique (URT) in a dual luciferase assay where K48-linked ubiquitin is tagged with renilla luciferase (RL) and a substrate for the E3 ligase is tagged with firefly luciferase (FL). The activity of the E3 ligase is determined via examination of the FL/RL ratio (Tian et al., 2019). This ratio can be used to identify inhibitors of E3 ligase activity (increase in FL/RL ratio) as well as the presence of E3 ligase activators (decrease in FL/RL ratio).

### Determination of E3 Ligase Substrates

The AD biology is complex, making it essential to understand the native interactions and substrates of E3 ligases in hopes of identifying therapeutic intervention modalities and effective small molecule ligands. Methods that enable identification of E3 ligase substrates include proteomics (to evaluate ubiquitination or PPIs), yeast two-hybrid (Y2H) assays, and *in vitro* phage display screening, among other techniques (Iconomou and Saunders, 2016).

Quantitative proteomics can be performed to identify the substrates of E3 ubiquitin ligases. Lee et al. (2011) reported the use of immunoaffinity enrichment of all ubiquitinated proteins from cells expressing His-tagged ubiquitin, followed by elution through an immobilized metal affinity chromatography (IMAC) chemical affinity column that is specific for His-tagged ubiquitin. The proteins were then separated via SDS-PAGE, and the bands digested and analyzed by liquid chromatography-mass spectrometry with tandem mass spectrometry (LC-MS/MS) (Lee et al., 2011). Determination of ubiquitination changes was carried out using stable isotope labeling by amino acids in cell culture (SILAC) methods, with the HA-ubiquitin negative control expressed in light medium and the His-ubiquitin in heavy SILAC conditions. Increased expression of ubiquitinated proteins in the heavy SILAC conditions were observed for substrates of the E3 ligase. As part of an orthogonal approach, Lee et al. (2011) also employed a three-way SILAC with a control siRNA compared to two HRD1 siRNAs, with the expectation that HRD1 substrates would have decreased ubiquitin levels. They observed peptides after a tryptic digestion step. Overall, approximately 400 ubiquitinated proteins and 1800 ubiquitinated peptides were identified for HRD1 using this strategy.

Martinez et al. (2018) highlight in their work a method to identify Parkin and Ube3A E3 ligase substrates using a ^bio^Ub (biotinylated ubiquitin) strategy in *Drosophila* neurons (Ramirez et al., 2018). Developing as well as mature neurons in *Drosophila* have been employed. This strategy relies on *Drosophila* engineered to express an *Escherichia coli* biotin ligase (BirA) that can biotinylate ubiquitin in photoreceptor neurons and result in incorporation of ^bio^Ub. Overexpression of the WT and mutant inactive negative control E3 ligases, such as Parkin and Ube3A, in the fly neurons is required. A pulldown of ^bio^Ub purified with neutravidin beads is followed by MS analysis that enables identification of substrates of the E3 ligases. While Ube3A was found to be active in both developing and mature *Drosophila* neurons, Parkin was only active in adult neurons. Ramirez et al. (2018) identified 79 substrates of Ube3A with increased ubiquitination upon overexpression of Ube3A in *Drosophila* neurons. Ube3A substrates included proteasomal proteins, Rpn10 and Uch-L5, as well as others that implicated a role of Ube3A in neuronal morphogenesis and synaptic transmission. Parkin substrates included proteins related to mitophagy, endocytic trafficking, ER stress, immunity, and apoptosis. Interestingly, the PINK1-mediated Parkin activation mechanism is not present in this model system, potentially indicating alternative ways to activate Parkin. Although these data suggest novel substrates for E3 ligases, follow-up studies in human neurons will be necessary.

More recently, Ordureau et al. (2018) used a quantitative proteomics approach called Parkin target-parallel reaction monitoring (Pt-PRM) to characterize Parkin substrate ubiquitination on the mitochondrial outer membrane (MOM) in neuronal cells. The Pt-PRM methodology allowed examination of kinetics, site specificity, and stoichiometry in parallel (Ordureau et al., 2018). Quantitative analysis of endogenous Parkin substrate ubiquitination in human embryonic stem cell (hESC)-derived NGN2-induced neurons (iNeurons) and DA neurons versus in HeLa cells was executed. In neurons expressing endogenous levels of Parkin, chain linkage analysis suggested little K48-linked chain production. This was not surprising given that K63-linked chains are predominantly linked to Parkin-mediated mitophagy. Finally, in neurons and HeLa cells no significant reduction in the 15 MOM Parkin substrates used in this study was observed at either a 1 or 4 h timepoint after depolarization.

An engineered protein technique using ubiquitination-activation interactions traps (UBAIT) employ a ubiquitin fused to a particular E3 ligase to detect the substrates of that E3 ligase (O’Connor et al., 2015; Zhao et al., 2020). The substrate binds to the target and forms an amide-linked E3-Ub-target complex that can be identified via proteomics analysis. This approach has proven applicable to HECT and RING E3 ligases, including the HECT E3 ligase Itch. A benefit of this approach is that it can capture relatively weak interactions.

Yeast two hybrid approaches can be used to identify PPIs in live yeast cells. A transcriptional factor such as Gal4 and a reporter gene are expressed, and the target protein is attached to a DNA binding domain that binds to Gal4 (Iconomou and Saunders, 2016). The putative binding partner is attached to a transcriptional activation domain so that if the target protein and the binding partner interact, then transcription of the reporter occurs. This approach was used with NEDD4 via cloning NEDD4 cDNA to a Gal4 binding domain and a cDNA library cloned with the Gal4 activation domain pGAD10 (Murillas et al., 2002). N4BP1 was identified as a monoubiquitylation nuclear protein, while NFBP3 was found to co-localize with NEDD4 in cytoplasmic vesicles. Alternatively, orthogonal ubiquitin transfer (OUT) techniques in yeast also enable identification of substrates via engineering the yeast cell surface display so that an E3 ligase can only transfer affinity-tagged ubiquitin variants (Wang et al., 2017). Wang et al. (2017) applied this approach to Ube3A and determined substrates including MAPK1, CDK1, CDK4, PRMT5, UbxD8, and β-catenin.

### Phage Display Assays to Identify Novel E3 Ligase Ligands or Substrates

As a methodology to enable identification of E3 ligase ligands, phage display is a biological strategy in which UbVs are generated that bind to E3 ligase Ub-binding domains or catalytic domains. These UbVs can inhibit HECT, monomeric RING/Ubox, or multi-subunit SCF RING E3 ligases or deubiquitinases. Alternatively, they can activate HECT or homodimeric RING E3 ligases (Ernst et al., 2013; Liu et al., 2021). In the case of RING/Ubox E3 ligases, inhibition occurs via blocking the E3-ubiquitin binding surface and activation occurs via stabilization of the E2-ubiquitin interaction (Gabrielsen et al., 2017). Zhang C. W. et al. (2016) demonstrated that with HECT E3 ligases, the UbV-based inhibition is due to blocking the E2 binding site, whereas activation is observed in response to UbV binding to the N-lobe exosite. Activation is suggested to be the result of release from an autoinhibited state (Zhang C. W. et al., 2016). Ordureau et al. (2018) developed a UbV that selectively inhibits Parkin^295–465^ and Parkin^410–465^ from binding to HECT E3 ligases (EDD1, HUWE1, WWP2, and HERC2) as well as non-specific controls (GST and BSA). The development of UbVs for phage display that can bind to domains of E3 ligases to elicit either inhibition or activation of function is a promising step in the pursuit small molecule ligands that would have potential utility in AD.

To screen for E3 ligase substrates, in a similar fashion to yeast cell surface display techniques, phage display techniques such as biopanning rely on the binding affinity of a protein library to immobilized receptors (Zhao et al., 2020). A protein library is fused to a protein (pIII) that is on the phage and, upon binding of the protein to immobilized receptors, a high-affinity phage-protein pair can then be selected and isolated. Bhuripanyo et al. (2018) used phage display techniques to engineer E2-E3 pairs compatible with the previously described OUT technique in which ubiquitin cascades only incorporate mutant ubiquitin. This strategy enabled identification of CHIP substrates, such as β-catenin and CDK4 (Bhuripanyo et al., 2018). Bhuripanyo et al. (2018) suggest that ER stress leads to the degradation of CDK4 via ubiquitination and proteasomal degradation, which could be mediated by CHIP.

The growing number of protein engineering and proteomics techniques should make the elucidation of the substrates of E3 ligases implicated in AD feasible. In turn, a better understanding of the complex biological functions of these E3 ligases may aid the development of novel chemical entities to modulate their function(s).

## Future Directions

### Proteolysis Targeting Chimeras for Alzheimer’s Disease

Proteolysis targeting chimeras (PROTACs) are heterobifunctional molecules that contain a ligand for an E3 ligase and a ligand for a POI attached via a linker region (Pettersson and Crews, 2019). The PROTAC brings the E3 ligase into close proximity to the POI via formation of a ternary complex, which facilitates the transfer of a ubiquitin from the Ub-E2-E3 complex onto a lysine residue on the POI. Attachment of ubiquitin to the POI as K48-linked polyubiquitin chains induces 20S proteasomal degradation of the POI and releases the PROTAC to degrade more POI. This catalytic mechanism of degradation of the POI means that lower and/or less frequent doses of PROTACs are often needed (Mares et al., 2020). In addition, the effect of degradation as opposed to inhibition can often lead to enhanced or longer-lived cellular effects when dosing with PROTACs (Wang and Zhou, 2018; You et al., 2020). PROTACs have recently proven effective in the clinic with androgen and estrogen receptor targeting PROTACs for cancer (Mullard, 2019).

Despite the success of PROTACs, there is still only a paucity of ligands available for E3 ligases (Ishida and Ciulli, 2021). Additional opportunities for the development of ligands for E3 ligases include tissue-specific E3 ligases that could elicit targeted degradation effects within the body. The development of novel ligands for E3 ligases presents the opportunity to develop PROTACs that degrade undruggable proteins within the proteome that are not currently targeted via available E3 ligases. Furthermore, new E3 ligase ligands could provide variable degradation potential of highly homologous proteins within the same family, offering enhanced selectivity versus traditional inhibition (Smith et al., 2019).

**FIGURE 5:**
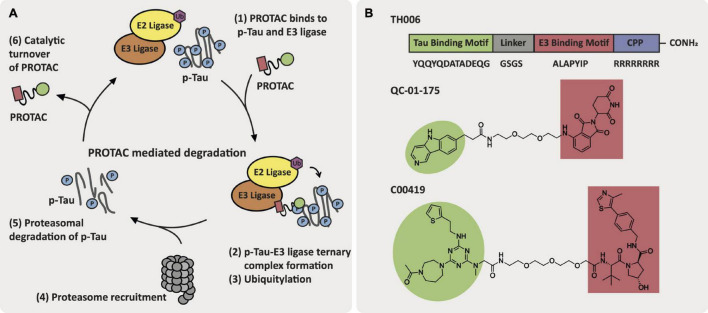
AD-relevant tau-targeting PROTAC approaches. **(A)** Key events in the PROTAC-mediated degradation cycle. **(B)** Structures of tau-targeting PROTACs TH006, QC-01-175, and C00419.

Current approaches toward PROTAC-mediated degradation within AD are focused on the targeted degradation of tau and hydrophobic tagging of tau that does not rely on E3 ligase recruitment (Gao et al., 2017b; Hyun and Shin, 2021). The first reported tau-degrader, TH006, by Chu et al. (2016) was a peptidic-based PROTAC with a cell penetrating peptide motif attached. TH006 reduced tau levels and improved viability in Aβ-treated primary neuron cells and in 3xTg-AD mice, which harbor three mutations associated with familial AD and exhibit both tau and Aβ pathology. In a similar approach, Lu et al. (2018) developed a tau-targeting PROTAC that contained a peptidic ligand for the E3 ligase Keap-1. This PROTAC induced tau degradation in SH-SY5Y, Neuro-2a, and PC-12 tau-overexpressing cell lines. Silva et al. (2019) adapted this strategy to develop a PROTAC, QC-01-175, that is comprised of a positron emission tomography (PET) probe appended to a cereblon (CRBN) E3 ligase ligand via a PEG2 linker. QC-01-175 induced a reduction in levels of tau and hyperphosphorylated tau (p-tau S396, S404) in human neuronal cell models derived from PSP patients with a tau-A152T risk variant and behavioral-variant FTD patients with a tau-P301L autosomal dominant heterozygous mutation. Additionally, QC-01-175 can differentiate between the disease-associated tau and normal tau in iPSC-derived neuronal cell models. Global proteomics analysis following 4 h treatment with 1 μM of QC-01-175 in A152T neurons demonstrated off-target degradation of the proteins ZFP91, ZNF653, and ZNF827, which are known immune-modulatory drug targets of CRBN. Finally, Wang et al. (2021) reported a tau degrader, C00419, that contains a VHL E3 ligase recruiter (VH032-derived ligand) and a tau ligand connected via a PEG3 linker region. C00419 was able to clear tau *in vitro* in SH-SY5Y cells as well as in HEK293 cells either stably or transiently transfected with hTau and *in vivo* in wild-type, 3xTg and hTau mice (Wang et al., 2021). Spine density was measured in neurons from 3xTg mice following subcutaneous administration of multiple doses of C004019 (3 mg/kg), which resulted in an increase in spine density. Similarly, in hTau mice the spine density and neurite arborization in the hippocampal dentate gyrus increased following subcutaneous administration of multiple doses of C004019 (3 mg/kg). Structures of these promising tau-targeting PROTACs are featured in Figure 5.

The development of tau-targeted PROTACs has pioneered the idea that PROTACs can directly induce degradation of key components that drive AD pathology. The generation of Aβ peptides from the cleavage of APP and accumulation of Aβ in AD is a classic hallmark of the disease. Despite knowledge of the amyloidogenic pathway, clearance of Aβ has proven a challenging therapeutic direction and there is not currently a small molecule strategy that allows direct binding and modulation of Aβ. As PROTACs are being used to target ‘undruggable proteins,’ eliciting Aβ degradation via a PROTAC offers a way to clear these pathogenic accumulations and could represent a viable strategy in AD (Gao et al., 2017a). The availability of Aβ PET ligands could enable a similar strategy to that employed by Silva et al. (2019) with respect to tau, in which Aβ PET ligands could be converted to Aβ-targeting PROTACs. Additionally, PROTACs targeting overexpressed or overactive proteins implicated in the pathogenesis of AD for degradation could lead to the regulation of novel pathways within AD. DUBTACs, conceptually similar to PROTACs in that both approaches utilize heterobifunctional molecules, is an emerging strategy that leads to deubiquitination of proteins, and has potential to further enable new therapeutic directions in AD (Henning et al., 2021). DUBTACs offer the ability to stabilize proteins that are otherwise downregulated in AD, thus providing new and different disease modifying strategies versus inhibition or degradation. PROTAC development within neurodegeneration is dependent on the generation of ligands for the desired target for degradation, and generation of ligands for the E3 ligases to be harnessed. Although tissue-specific and/or disease specific E3 ligases that could be targeted via PROTACs have been identified, the lack of available small molecule ligands impedes their development and evaluation of these therapeutic hypotheses. A paucity of small molecule ligands is available for the dysfunctional AD E3 ligases mentioned herein this review. A key first step toward enhanced understanding of the underlying disease pathology of E3 ligases in AD is the design of high-quality chemical probes to assess the effects of either inhibition, activation, or degradation of these E3 ligases on the key phenotypes of AD in neurons.

### Ubiquitin Proteasome System Dysfunction in Amyotrophic Lateral Sclerosis and Parkinson’s Disease

The dysfunctional role of UPS also drives the pathogenesis and regulation of aggregated proteins in neurodegenerative diseases such as amyotrophic lateral sclerosis (ALS) and Parkinson’s disease (PD). Since UPS dysfunction is exacerbated by protein aggregation, it contributes to the neuronal dysfunction and/or loss that plagues patients. Specifically, in PD the accumulation of α-synuclein (α-syn) generates Lewy Bodies that can hamper synaptic function via impaired UPS, reduced autophagy, and damaged lysosomes (Guerroué and Youle, 2021). Within ALS, protein aggregates of TAR DNA-binding proteins 43 (TPD-43), superoxide dismutase type-1 (SOD1), and RNA-binding protein fused in sarcoma (FUS) are linked to mitochondrial dysfunction and degeneration, impaired UPS, and reduced autophagy (Chisholm et al., 2021). Farrawell et al. (2020) identified UPS genes that are either up or downregulated in ALS neurons, noting a predominant decrease in the expression of UPS genes in ALS. Despite this, several E3 ligases are upregulated in ALS-afflicted neurons. Some of the same E3 ligases discussed with respect to AD that could represent therapeutic targets for AD also play essential roles in the pathology of PD and/or ALS. Known exogenous ligands that were included in the AD-specific sections may find utility in treating these other neurodegenerative diseases.

#### NEDD4-1

The E3 ligase NEDD4-1 is upregulated in AD and ALS and its expression is variable in PD when examining human patient brains and spinal cord samples (Kwak et al., 2012). In PD patients, NEDD4-1 is expressed in neurons that contain Lewy Bodies and ubiquitinates α-syn via K63-linked ubiquitin, leading to α-syn degradation via the lysosomal pathway (Tofaris et al., 2011; Huang et al., 2019; Farrawell et al., 2020). In rat primary cortical neurons that model PD, NEDD4-1 co-localizes with and decreases the expression of RTP801, which is an elevated protein in PD that triggers neuronal death (Canal et al., 2016). Although degradation of α-syn and RTP801 would be beneficial in alleviating PD, NEDD4-1 is also downregulated in nigral neurons from human sporadic PD patient brains. Thus, strategies to increase activity and/or upregulate NEDD4-1 E3 ligase are required in the context of PD. Although it is clear that NEDD4-1 is upregulated in ALS, the exact role in disease pathogenesis is unknown.

#### TRAF6

In the context of ALS, TRAF6 is localized to the mitochondria in the spinal cord of rats and interacts with mutant SOD1 variants SOD1^*A*4*V*^ and SOD1^*V*148*G*^ leading to polyubiquitination and/or aggregation of SOD1 that results in cellular accumulation (Semmler et al., 2020). Interestingly, Semmler et al. (2020) reported that a decrease in TRAF6 leads to a reduction in mutant SOD1 aggregation and an increase in turnover independent of TRAF6 E3 ligase activity. Chung et al. (2013) demonstrated that expression of TRAF6 in PD is inversely increased in human tissues when compared to Parkin. Parkin is responsible for the reduction of TRAF6 levels via proteasomal degradation and Chung et al. (2013) demonstrated that the loss-of-function Parkin mutations (R42P and T240R) did not result in decreased expression of TRAF6. This suggests that increased Parkin E3 ligase activity or a decrease in TRAF6 E3 ligase levels would be beneficial to PD patients.

### Proteolysis Targeting Chimeras Targeting Removal of Protein Aggregates in Parkinson’s Disease and Amyotrophic Lateral Sclerosis

Aggregated proteins are a problem common to several neurodegenerative diseases, including AD, PD, and ALS. The use of PROTACs to target these misfolded and/or aggregated proteins for degradation is a viable therapeutic approach. As an example, tau-directed PROTACs as potential AD therapeutics were discussed in a previous section.

Several strategies have been employed to target α-syn for proteasomal and lysosomal degradation. Firstly, Fan et al. (2014) reported a synthesis of a peptide containing an α-syn-binding sequence, cell penetrating peptide (CPP) component, and an autophagy-targeting motif that induced α-syn degradation in primary cultured rat neurons. Although this strategy was effective in their cellular model, lysosomal impairment is a common hallmark of PD that is responsible for accumulation of α-syn and which may reduce the efficacy of this and related approaches (Navarro-Romero et al., 2020). Qu et al. (2020) designed a peptide-based PROTAC with a TAT-protein binding domain-proteasome targeting motif (TAT-PBD-PTM), containing a TAT CPP that simultaneously binds to intracellular α-syn and E3 ligases to induce α-syn proteasomal degradation. The PTM is comprised of a small tetrapeptide with the amino acid sequence RRRG, which was previously identified as a degron that induces proteasomal degradation (Bonger et al., 2011). Via mass spectrometry Qu et al. (2020) identified that this peptide region binds to E3 ligases TRIP12, STUB1, and UHRF1. Furthermore, significant degradation of α-syn was observed in SK-N-SH cells and in primary cortical neurons following treatment with TAT-PBD-PTM (25 μM). This peptide-based PROTAC induced a decrease in endogenous α-syn by >70% in primary neurons that were cultured for 8 days with treatment of TAT-PBD-PTM at 25 μM for 4 h. Finally, the PROTAC-based company Arvinas has patented PROTACs targeting α-syn that employ small molecule E3 ligase ligands for VHL and CRBN appended to α-syn-targeting compounds. These PROTACs result in <35% α-syn after treatment at 1 μM for 48 h in HEK293 TREX α-syn A53T cells (Kargbo, 2020).

To our knowledge, there are currently no reported ALS relevant PROTACs, which could be used to target protein (mutant SOD-1 or TDP-43, for example) aggregates found in ALS patients. Rao et al. (2021) summarized the current landscape of TDP-43 ligands for the different domains of TDP-43. Solved crystal structures of TDP-43 have enabled the development of TDP-43 ligands. These ligands consist of small molecules, peptides, and oligonucleotides and could be applied to the development of PROTACs akin to the tau-targeted degraders that utilize known PET tracers for tau. Ligands of SOD-1 have not been described in the literature, precluding SOD1-targeting PROTAC development at this point.

The development of novel E3 ligase ligands that have disease-specific functions or tissue-specific locations could greatly enable the development of PROTACs aimed at treating certain neurodegenerative diseases. The initial success of PROTAC technology for the degradation of aggregated and/or misfolded proteins in neurodegenerative diseases demonstrates that further development in this area could be therapeutically beneficial in diseases such as AD, PD, and ALS. This is a promising future direction of the PROTAC and neurodegenerative fields.

## Author Contributions

FP and AA wrote various sections and provided extensive edits to the final document. Both authors contributed to the article and approved the submitted version.

## Conflict of Interest

The authors declare that the research was conducted in the absence of any commercial or financial relationships that could be construed as a potential conflict of interest.

## Publisher’s Note

All claims expressed in this article are solely those of the authors and do not necessarily represent those of their affiliated organizations, or those of the publisher, the editors and the reviewers. Any product that may be evaluated in this article, or claim that may be made by its manufacturer, is not guaranteed or endorsed by the publisher.
